# Indicators of immunosuppression peripartum in dual purpose cows in the tropics affected health, productive and reproductive parameters

**DOI:** 10.1590/1984-3143-AR2021-0040

**Published:** 2022-01-07

**Authors:** Miguel Ángel Lammoglia, Ivan Avalos, Amalia Cabrera, Maria Rebeca Rojas, Nora Garcez, Abigail Tabarez

**Affiliations:** 1 Programa Educativo de Medicina Veterinaria y Zootecnia, Facultad de Ciencias Biológicas y Agropecuarias, Universidad Veracruzana, Tuxpan, Veracruz, México

**Keywords:** neutrophils, β-hydroxybutyrate, glucose, endometritis, tropics

## Abstract

The objective of the study was to identify immunosuppression peripartum indicators in dual purpose cows in the tropics and determine their effects on productive and reproductive parameters. The indicators used were: changes in leukocyte and neutrophils population, concentrations of energy metabolites (β-hydroxybutyrate and glucose) and body condition scores (BCS). Blood sampling and BCS (scale 1 – 5) were taken weekly during the peripartum. Uterine health was assessed (3 weeks postpartum) by ultrasonography and using a vaginal score (0-3) described by Sheldon et al. (2006). Cows (n=30) were classified as healthy or clinical endometritis (CE). CE prevalence was as high as 29.6%. Leukocyte and neutrophils populations diminished while in the peripartum and were lower (P<0.05) in cows suffering CE. Healthy cows had higher (P<0.05) daily milk production than those with CE (18.84±0.63 vs 14.76±0.84 kg). CE cows had lower (P<0.05) reproductive performance compared with healthy cows (open days: 244.40 ± 35.00 vs 178.00 ± 23.33 and services by conception 3.33 ± 0.51 vs 1.83 ± 0.34). BCS similarly (P>0.05) decreased following parturition in both groups. Concentrations of energy metabolites during peripartum fluctuated in a similar (P>0.05) manner in healthy and CE cows. In summary, dual purpose cows in tropical conditions, presented peripartum immunosuppression indicators, characterized by a decline in the leukocyte population, mainly neutrophils, as well as decreased glucose concentrations and BCS postpartum. In addition to it, there was a rise in the β-hydroxybutyrate concentrations and cows presenting CE had a negative effect in the productive and reproductive parameters.

## Introduction

Milk production increased at a global level and went from 530 million tons in 1988 to 843 million in 2018 which represent almost a 60% increase in 30 years. It is also estimated that nearly 150 million families are involved in its production. Most of the production units (75%) are in developing countries, and these generate 26% of the milk of the world. However, the remaining 25% are in developed countries and produce 74% of the milk (Ramirez-Rivera et al., 2019).

Productivity may increase in the tropics over the coming years due to the high demand for generating in a more sustainable and environmentally – friendly way. There are large extensions of pastures in the tropics under intensive rotational grazing management that contribute to deposit vast amounts of carbon on earth and mitigate the greenhouse effect (Soussana et al.*,* 2010). Furthermore, most of the bovine animal population is in said area, which characterizes by having various production systems, but particularly the production system known as dual purpose (Vilaboa et al.*,* 2009). The aforementioned system consists of producing milk and selling the calves past weaning (Ramirez-Rivera et al.*,* 2019). Even though the majority of the cows in tropics have certain degree of heterosis (*Bos indicus* x *Bos taurus*) and are less productive, these can be exposed to undergo the same physiological peripartum conditions (immunosuppression, negative energetic balance, anestrus, etc.), than specialized high – production cows.

Health issues during the peripartum period include infectious (placenta retention, metritis, and mastitis) and metabolic diseases (hypocalcemia, Ketosis, and dysplasia of the abomasum) which affect a great percentage of cows (LeBlanc, 2010). The infectious diseases are strongly related with immunosuppression (Aleri et al., 2016), high levels of ketone bodies (Ingvartsen and Moyes, 2013) which negatively affect the productive and reproductive parameters (Sordillo et al.*,* 2009; Giuliodori et al., 2013). The aim was to identify the presence of immune suppression indicators during the peripartum period of dual purpose cows in the tropics and evaluate their effects on productive and reproductive parameters.

## Materials and methods

The study was conducted in a tropical region with an elevation of 10 meters over the sea level, an average temperature and yearly annual precipitation of 24.9 °C and 1241 mm, respectively. Rainfall is plentiful during the summer and in the beginning of fall, with greater intensity from November through May.

All the animals were treated in strict accordance with Mexican laws for animal welfare and experimentation, the protocol were evaluated and approved with registration number COBIBA/003/2019 by Bioethical Committee of the Faculty of Biological and Agricultural Sciences, Veracruzana University.

Multiparous cows (n= 30; *Bos taurus x Bos indicus*) managed in an intensive grazing rotational system of Brizantha (*Brachiaria brizantha*) and African star (*Cynodon plectostachyus*) pastures were used. Furthermore, cows were given orange silage (10 ± 1.5 kg/cow/day), mineral salts, and free access to fresh water.

Blood samples were taken on a weekly basis, starting one week before the expected calving date, up until twelve weeks after parturition, in tubes with EDTA (4 ml, BD Vacutainer^®^, Becton, Dickinson and Company, Plymouth, United Kingdom) from the sanguineous plexus at the base of the tail. Glucose and β-hydroxybutyrate concentrations were established with fresh blood at the moment of extraction, and the leukocytes and neutrophils count were determined with blood stored at 4º C, for 48 hours.

The β-hydroxybutyrate concentrations were ascertained by using a cytometer of human use (FreeStyle^®^, Optium Neo™, Abbott, Oxfordshire, United Kingdom). 50 μl of blood was placed on the cytometer strip and the value displayed on the screen (mmol/l; Fiorentin et al., 2017). Similarly, concentrations of glucose were also determined with a glucometer of human use (OneTouch^®^, UltraMini™, LifeScan Inc, Milipitas, California, United States); 50 μl of blood were placed on the glucometer strip and its value (mg/dl) was recorded on the screen (Castillo-Valeriano et al., 2018).

Leukocyte and neutrophil counts were calculated as follows: stored blood was retrieved from the test tube at 4 °C with the Thoma pipette for white blood cells, up to the 0.5 mark. Then filled up to the 11 mark with Turk solution and both pipette outlets sealed, to homogenize for 3 minutes. On completion, the first three drops were discarded, and the fourth drop inserted into the Neubauer chamber, which sat for a minute until the solution and cells settled. Leukocyte counting took place and the number of found cells in all four quadrants were added, for subsequently multiplying by 50 (factor) to obtain the number of leukocyte cells per cubic millimeter of blood (Islam et al., 2014).

A blood smear was performed and dyed with a Hemacolor® rapid staining solution, to proceed with the differential leukocyte cell count. The recount of various leukocyte cells that were observed in blood smear was conducted with the cell counter, to reach the 100 cells. Once the percentage of neutrophils was attained out of the differential recount of cells (neutrophil amount on band is added and these are segmented so as to reflect the total percentage of the foregoing in the white blood cells) and the number of leukocytes derived from the recount in the Neubauer chamber, the absolute value of neutrophils per cubic millimeter of blood was obtained, through the rule of three.

Uterus health was assessed by ultrasound 21 ± 4 days, following parturition. Also, by scoring a sample collected manually from the vagina. The procedure to take the sample was to clean the vulva region with water and a hypoallergenic soap. A new palpating glove and lube was used per each cow to collect the sample. The sample collected was scored as clinical endometritis using the scale 0 – 3, as described by Sheldon et al. (2006). Score 0, contained mainly clear or translucent mucus, score 2, contained < 50% purulent material and score 3, contained more than 50% purulent material and occasionally sanguineous. During this study, clinical endometritis was considered only in cows that presented stage 3. All the cows that presented dystocia and/or fetal membranes retention during the time of the experiment were rejected for the study.

Body condition score was established on the same days in which the blood was drawn (1 week prior the delivery and 12 weeks post-partum). The used scale from 1-5, was described by Ferguson et al. (1994), in which body condition 1 is a cow with cachexia and a cow with body condition of 5, is an obese cow.

Milk was weighed every 7 days in Waikato milk meters integrated into the milk line. Milk and reproductive data were registered in the Vampp Bovino^®^ v.3.0 software (National University of Costa Rica, Heredia, Costa Rica) to later export the data into an Excel spreadsheet and run the statistical analysis. This was performed by using the STATISTICA v.7.0^®^ software (StatSoft, Tulsa, Oklahoma, United States) with the ANOVA models.

## Results

**Figure 1:**
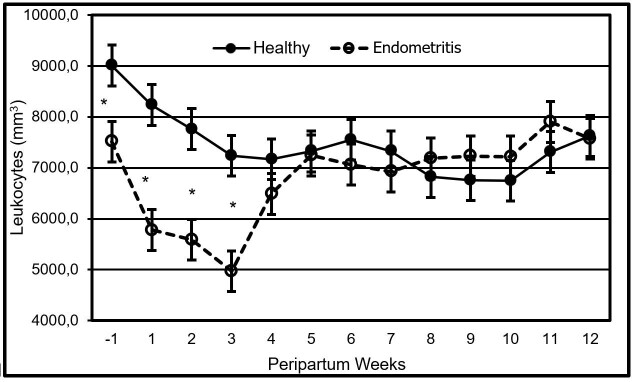
Effect of the peripartum week variable in uterine health over the leukocyte population in dual purpose cows. *Indicate that the data is statistically significant between groups (p<0.05).

The leukocyte population decreased in the week prior to calving up to the third postpartum week in all cows. However, cows diagnosed with clinical endometritis had a lower population of leukocytes and presented a further decline (p<0.05) in it. The same reached a lower count in the third week postpartum (Figure 1), in contrast with the healthy cows.

**Figure 2:**
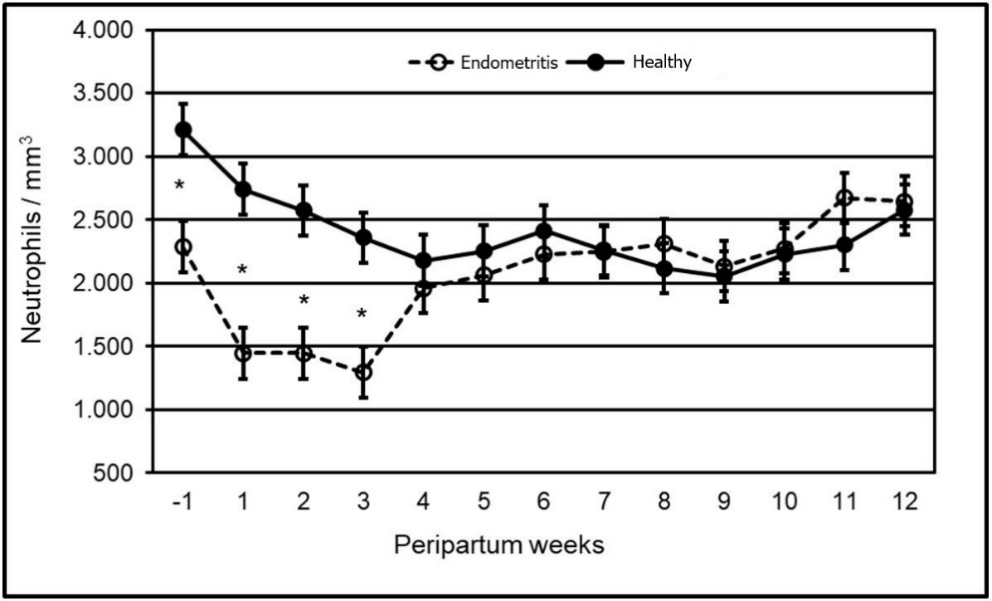
Effect of the peripartum week variable in uterine health over the neutrophil population of dual-purpose cows. *Indicate that the data is statistically significant between groups (p<0.05).

Similarly, the neutrophil population declined in one week prior to parturition, up to the third postpartum week in all cows, again observed that the cows that developed clinical endometritis postpartum presented a lower (p<0.05) neutrophil population and a higher decrease in it during the 1–3 weeks of peripartum, in contrast with the healthy cows (Figure 2).

The concentrations in blood of glucose and β-hydroxybutyrate were alike (p>0.05) among healthy cows and those diagnosed with clinical endometritis (Table 1). However, when analyzing the metabolites according to peripartum, it was observed that glucose concentrations were significantly higher prior the delivery, rather than after it. In contrast, β-hydroxybutyrate concentrations were significantly lower during the prepartum period than in postpartum (Table 1).

**Table 1:** Variations in the glucose concentrations and the β-hydroxybutyrate in blood during the peripartum in dual purpose cows.

**Energy Metabolites**	**Peripartum**	
	**Prepartum**	**Postpartum**
**Glucose (mg/dl)**	40.4 ± 2.0^a^	35.4 ± 0.7^b^
**β-hydroxybutyrate (mmol/l)**	0.9 ± 0.4^a^	1.6 ± 0.2^b^

Different letters for each variable indicate differences (*p*<0.05).

**Figure 3:**
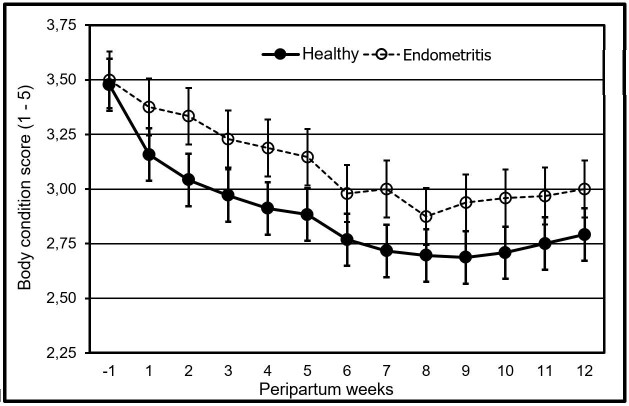
Changes in the body condition score (scale 1–5) during the peripartum in healthy dual purpose cows, and cows diagnosed with metritis as well (p>0.05).

Body condition scores (BCS) changed (p>0.05) overtime. BCS similarly (p>0.05) decreased following parturition in both groups (Figure 3).

In terms of productivity, the cows that stayed healthy had a better (p<0.05) daily average production and reached higher levels of production once the 100 days of lactation have been met, in comparison with cows that were diagnosed with clinical endometritis (Table 2). One of the biggest impacts was in the area of reproduction; cows suffering clinical endometritis had a lower (p<0.05) productive performance when days to the first service, days open, and services for conception were incremented (Table 2).

**Table 2:** Productive and reproductive parameters of healthy dual purpose cows and cows diagnosed with endometritis.

**Parameter**	**Healthy**	**Endometritis**
**Average daily milk (kg)**	18.84 ± 0.63^a^	14.76 ± 0.84^b^
**Production on day 100 (kg)**	1934.88 ± 70.65^a^	1466.25 ± 105.98^b^
**Days on the first service**	76.73 ± 14.00^a^	143.90 ± 21.0^b^
**Open days**	178.00 ± 23.33^a^	244.40 ± 35.00^b^
**Services for conception**	1.83 ± 0.34^a^	3.33 ± 0.51^b^

Different letters for each parameter indicate differences (*p*<0.05).

## Discussion

One of the most common illnesses during postpartum in specialized dairy cattle are metritis and endometritis which mainly derives by immunosuppression. A high prevalence of these illnesses has severe productive and reproductive repercussions, thus, of economic nature. The results of this study with dual purpose cows in pasture within a tropical region indicate a high prevalence of clinical endometritis (26.9%) by comparison to the specialized dairy cattle which enables ranges from 10 – 20%. Even so, there is not enough published information regarding bovine animals of dual purpose in pasture within the tropics, to compare the results with. It is important to mention that uterine health assessment it is not a common practice in dual purpose cows, and it could be possible that many of these cows that had clinical endometritis might have lower pregnancy rates. Therefore, it could be recommended the necessity to include this practice in this dual purpose cow management.

The leukocyte and neutrophil population were affected (p<0.05) by the variables week, health, and week per health interactions. All cows, whether healthy or with clinical endometritis, presented a decline in the leukocyte and neutrophil population, that started one week before parturition up to three weeks postpartum. This behavior had been reported in dairy cows (McDougall et al.*,* 2017; Kimura et al.*,* 2014) but not in dual purpose cows in pasture, at the tropics. It might be suggested that the aforementioned cows undergo a reduction in the leukocyte and neutrophil populations (immunosuppression) similar to the dairy cows in confinement. However, the much – quoted populations in dairy cattle returned to the original counting 3 weeks postpartum (McDougall et al., 2017; Kimura et al., 2014), in contrast to the dual purpose cows in pasture, subject of the present study, which yet, on week 12 after calving did not reach the existent count prior the immunosuppression. This longer duration of low count of leukocytes and neutrophils in dual purpose cows in the tropic may be attributed to multiple factors. However, due to the fact that the main source of nutrients in this habitat is the fodder, which is highly variable in quantity and quality (Corro et al., 1999), this falls far short of the cow’s nutritional needs during the postnatal period. Thus, the negative energetic balance gets prolonged (Ingvartsen and Moyes, 2013) along with hypoglycemia and high concentrations of β-hydroxybutyrate (Castillo-Valeriano et al.*,* 2018); at the same time, such factors might have contributed to an extended immunosuppression (Esposito et al., 2014).

The leukocyte and neutrophil population diminished more in cows suffering clinical endometritis compared to healthy bovine animals. Results of this study were consistent with the reported data on specialized dairy cattle (Islam et al.*,* 2014; Bromfield et al., 2018) from which not only a decline in the population of neutrophils has been informed but also in their immunological capacity and still, a greater loss of such in the cows having metritis (McDougall et al., 2017; Kimura et al.*,* 2014).

During this study, one week prior to calving, cows diagnosed with clinical endometritis started having lower populations of neutrophils and leukocytes than those that were healthy. This data is similar to that published on specialized dairy cattle, where there is a lower population of neutrophils and leukocytes in cows that developed metritis (Hammon et al., 2006). All cows suffer immunosuppression in the peripartum but their ability to balance metabolic changes at this stage will largely determine the degree of immunosuppression (Nonnecke et al., 2003). It is possible that in this study, grazing dual purpose cows that developed clinical endometritis had difficulty stabilizing some metabolic changes before the delivery and therefore, accentuated the immunosuppression that unleashed the clinical endometritis.

Unlike healthy cows, Cui et al. (2019) larger populations of neutrophils were reported in cows diagnosed with metritis. Also, Islam et al. (2014) reported greater percentages of neutrophils in the latter. Findings from both these studies are not in line with the results of this study conducted on dual purpose cows in pasture at the tropics, nor with the results of confined with confined dairy cows. As for the metritis diagnosis, it can be said that this has been a subject of much controversy for several years since grades of the disease have been suggested (Sheldon et al., 2006), and a significant fact of this, is the day on which the diagnosis is made after the postpartum (LeBlanc et al., 2002). On this regard, it bears mentioning that not all researchers consider the above for the statistical analysis. In this study in particular and others that had been released on specialized dairy cattle, Sheldon et al. (2006) reported classification has been taken into consideration. The latter, accounts 3 different degrees of endometritis (0= clear mucus (healthy); 1= mucus that contains white or pale white mucus; 2= discharge which contains 50% of mucopurulent whitish material; 3= discharge which contains 50% white or yellow purulent material and / or blood).

Metritis definition reported by Drillich et al. (2001), was contemplated in the studies conducted by Islam et al. (2014) and Cui et al. (2019). In such, a cow with metritis is one that, when palpated via transrectal thick walls of the uterus are noted, along with high rectal temperature (≥ 39.5 °C), and a fetid discharge. Consequently, it be said that in these 2 studies, cows with grade 3 without symptomatology, cows with grade 2 and 1 remained in the healthy cow group. In other words, only cows clinically diagnosed with endometritis were taken into consideration, and this could have made a significant difference in the results shown in this research conducted on dual purpose cows in pasture at the tropic, as well as in the studies that used dairy cows in confinement.

The cows had a decline in their body condition as of the week prior to parturition until the week number eight postpartum. A similar outcome has been reported in cows of dual purpose of the tropic (Castillo-Valeriano et al.*,* 2018) when losing about one point of their body condition. When referring to dairy specialized cattle, the latter condition starts one week prior the delivery, in the peripartum period, with the lowest point in the week number 4 – 6 of postpartum (Wathes et al., 2007; Pires et al., 2013). This loss of body condition is largely due to the decline in consumption of dry matter (Ingvarsten and Moyes, 2015). A prolonged recovery period when losing the body condition once the delivery has taken place in grazing dual purpose cows in the tropic, may derive from the fact that the main source of nutrients is the fodder in the tropical habitat, which is highly variable in quantity and quality, as previously described (Corro et al.*,* 1999) and that it does not meet the nutritional needs of the cows during the postnatal period.

In this study, concentrations of β-hydroxybutyrate and glucose in blood in dual purpose cows in pasture within the tropic were higher before calving than the postpartum period, in both, healthy cows, and cows diagnosed with clinical endometritis. These results are similar to those reported in specialized dairy cattle (Zhang et al., 2019). It is important to mention that although the glucose and β-hydroxybutyrate patterns were alike, the glucose values in the blood were lower and the β-hydroxybutyrate higher in grazing cows of double purpose in the tropic compared to the confined dairy female bovine animals (Wankhade et al.*,* 2017). The initiation of lactation is a huge nutritional challenge, and in bovine animals and dual purpose cattle these needs are not contemplated. Like stated before, the cornerstone of diet in pasture is the fodder, which can only contribute little to the needs of a fresh cow. Hence, this cow has to draw on body reserves, fact that could mark the differences between the glucose concentrations in blood and the β-hydroxybutyrate compared with the specialized and confined female bovine animals.

Unlike this study, other research (McArt et al., 2012; Hammon et al.*,* 2006), reported that in fresh cows with infectious conditions like metritis, these presented various concentrations of glucose and β-hydroxybutyrate in blood, in contrast to healthy cows. Difference between results could have been due to the severity of the infectious process. Lochmiller and Deerenberg (2000) informed that acuteness of animal in the infectious process influences the amount of energy needed to stimulate the immune response, which generally requires 10 – 40% extra energy. Nevertheless, when it comes to extremely severe infections, a cow can require up to 2 kg of extra glucose in 24 hours to respond to the infection. In regards the present study, perhaps the endometritis degree was not severe enough to demand larger quantities of glucose and for this reason, there was similarity among the concentrations of these molecules between cows diagnosed with clinical endometritis and the healthy cows.

The cows suffering clinical endometritis had a lower production of milk compared to the healthy cows, which conformed with the majority of the studies consulted (Bromfield et al., 2018; Giuliodori et al.*,* 2013; Wittrock et al., 2011; Dubuc et al., 2011). These similarities can be explained by the fact that all cows with infectious processes reduce the consumption of the dry matter, which decreases the level of available energy for milk synthesis, in comparison with the healthy cows. However, other studies do not find effects of clinical endometritis over the production of milk (Markusfeld and Ezra, 1993; Goshen and Shpigel, 2006). During the course of these studies, acuteness of clinical endometritis was not reported and this happens to be relevant since the severity of it has a significant impact in the energy demand so that the immune system counterattacks, which affects the animal productivity (Sheldon et al., 2006; Lochmiller and Deerenberg, 2000).

The cows with clinical endometritis in this paper, had a larger number of open days than healthy cows, which is consistent with the various studies (Vallejo-Timaran et al., 2017; Toni et al.*,* 2015; Giuliodori et al., 2013). Likewise, cows with clinical endometritis had a greater interval from calving to its first service than healthy cows, which agrees with consulted literature (Elkjær et al., 2013; Toni et al., 2015). The number of services per conception were higher in cows with clinical endometritis than healthy cows. Comparable results were found in studies concerning dairy specialized cattle (LeBlanc et al., 2002; Gilbert et al., 2005; Barlund et al., 2008), in which it is evidenced that metritis increases the number of services per conception. Generally, one can say that clinical endometritis or metritis can affect reproductive performance (Giuliodori et al., 2013; Sheldon et al., 2006). Like other infectious processes, metritis stimulates an inflammatory response, which in this case, shows in the endometrium. Due to the peculiarity of the cow uterus – ovary circulatory system, the inflammatory process spreads to the ovary causing oophoritis and deteriorating the fertility of oocytes for weeks (Sheldon et al., 2019). The combination of these factors causes low reproductive parameters in specialized dairy cattle, and the findings of this study may indicate the same effect in dual purpose cows in pasture, at the tropic.

## Conclusion

The results of the present study reveal that just like in specialized dairy cattle, there is peripartum immunosuppression indicators in grazing dual purpose cows in tropical conditions that appear due to the decrease in the leukocyte population (mainly neutrophils), a decline in the glucose concentrations, body condition and increase in the β-hydroxybutyrate. Also, in cows that showed clinical endometritis, the effects were stronger since these had lower productive and reproductive performance.
